# A Prognostic Model for Breast Cancer Based on Cancer Incidence-Related DNA Methylation Pattern

**DOI:** 10.3389/fgene.2021.814480

**Published:** 2022-01-03

**Authors:** Zhenchong Xiong, Lin Yang, Juan Ao, Jiarong Yi, Xiazi Zouxu, Wenjing Zhong, Jikun Feng, Weiling Huang, Xi Wang, Zeyu Shuang

**Affiliations:** ^1^ State Key Laboratory of Oncology in South China, Collaborative Innovation Center of Cancer Medicine, Department of Breast Oncology, Sun Yat-sen University Cancer Center, Guangzhou, China; ^2^ Department of Radiation Oncology, Nanfang Hospital, Southern Medical University, Guangzhou, China; ^3^ Department of Neurology, Guangzhou First People’s Hospital, Guangzhou, China

**Keywords:** breast cancer, prognosis, DNA methylation, immune, nomogram

## Abstract

Breast cancer (BC) is the most diagnosed cancer and the leading cause of cancer-related deaths in women. The purpose of this study was to develop a prognostic model based on BC-related DNA methylation pattern. A total of 361 BC incidence-related probes (BCIPs) were differentially methylated in blood samples from women at high risk of BC and BC tissues. Twenty-nine of the 361 BCIPs that significantly correlated with BC outcomes were selected to establish the BCIP score. BCIP scores based on BC-related DNA methylation pattern were developed to evaluate the mortality risk of BC. The correlation between overall survival and BCIP scores was assessed using Kaplan–Meier, univariate, and multivariate analyses. In BC, the BCIP score was significantly correlated with malignant BC characteristics and poor outcomes. Furthermore, we assessed the BCIP score-related gene expression profile and observed that genes with expressions associated with the BCIP score were involved in the process of cancer immunity according to GO and KEGG analyses. Using the ESTIMATE and CIBERSORT algorithms, we discovered that BCIP scores were negatively correlated with both T cell infiltration and immune checkpoint inhibitor response markers in BC tissues. Finally, a nomogram comprising the BCIP score and BC prognostic factors was used to establish a prognostic model for patients with BC, while C-index and calibration curves were used to evaluate the effectiveness of the nomogram. A nomogram comprising the BCIP score, tumor size, lymph node status, and molecular subtype was developed to quantify the survival probability of patients with BC. Collectively, our study developed the BCIP score, which correlated with poor outcomes in BC, to portray the variation in DNA methylation pattern related to BC incidence.

## Introduction

Breast cancer (BC), the most diagnosed cancer globally, is the leading cause of cancer-related deaths in women (Siegel et al., 2020). As the incidence of BC has continuously increased over recent years, BC has become a major public health problem, and one in eight women would be affected by BC by the age of 85 years in high-income countries (Britt et al., 2020). With a deep understanding of cancer biology, BC is subdivided into four molecular subtypes (luminal A, luminal B, HER2, and TNBC) based on the expression of the estrogen receptor (ER), progesterone receptor (PR), HER2 receptor, and Ki-67 (Abreu et al., 2020). Although BC therapy guided by molecular subtyping greatly reduces BC mortality, cancer recurrence inevitably occurs (Alwan and Tawfeeq, 2019). Investigations of prognostic methods for predicting outcomes in BC may provide clues for improving cancer treatment.

DNA methylation, an epigenetic modification, plays an important role in cancer development (Jones et al., 2016). Although previous studies have shown that BC is a genetic disease, genome-wide variations in DNA methylation have been observed in cancer cells (Flavahan et al., 2017; Widschwendter et al., 2018). DNA methylation induces chromatin structure changes and inhibits gene expression mediated by DNA methyltransferases. Aberrations in DNA methylation, which are affected by environmental, lifestyle-related, and heritable factors, may induce cancer development by silencing tumor suppressors and/or re-activating oncogenes (Baylin and Jones, 2011; Koo et al., 2015; Muvarak et al., 2016). In BC, hypomethylation of stemness- and proliferation-associated genes in circulating tumor cells promotes stemness and metastasis (Gkountela et al., 2019). Thienpont et al. indicated that tumor hypoxia induces hypermethylation and promotes BC progression by inactivating TET enzymes (Thienpont et al., 2016). Thus, tracking the changes in DNA methylation pattern associated with BC is helpful for developing prognostic methods for BC.

The purpose of our study was to discover DNA methylation pattern and develop a prognostic model based on BC-related DNA methylation pattern. We identified 361 breast cancer incidence-related probes (BCIPs) that were differentially methylated in blood samples from women at a high risk of BC and BC tissues. Twenty-nine of the 361 BCIPs that were significantly correlated with BC outcomes were included to establish the BCIP score. In BC, the BCIP scores were significantly correlated with malignant BC characteristics and poor outcomes. Furthermore, we assessed the BCIP score-related gene expression profile and observed that the BCIP score-related gene profile participated in the process of cancer immunity. BCIP scores were negatively correlated with immune cell infiltration and the immune checkpoint inhibitor (ICI) response in BC tissues. Finally, a nomogram comprising the BCIP score, tumor size, lymph node status, and molecular subtype was developed to quantify the survival probability of patients with BC.

## Materials and Methods

### Data Collection and Processing

For the GSE51057, GSE72308, and TCGA-BRCA DNA methylation datasets, genome-wide methylation data were profiled using Illumina Infinium HumanMethylation450 BeadChips Assay (Illumina 450 K platform). For the GSE57285 DNA methylation dataset, genome-wide methylation data were profiled using Illumina Infinium HumanMethylation27 BeadChips Assay (Illumina 27 K platform). The DNA methylation level of each probe was calculated using *β* values ranging from 0 (no DNA methylation) to 1 (complete DNA methylation). Probes containing missing values in over half of the samples in each dataset were removed, while missing values of the remaining probes were imputed with the k-nearest neighbors imputation method. Probes located on the sex chromosome and probes containing known single-nucleotide polymorphisms were removed (Price et al., 2013). Finally, 23,614 probes were selected for further investigation. The above process was performed using the R package Champ (Tian et al., 2017).

For gene expression data, mRNA expression data were obtained from the Cancer Genome Atlas (TCGA) database. Background correction and normalization of mRNA expression data were performed using the R package limma (Ritchie et al., 2015). Expression data for protein-encoding genes were included in further analysis.

### Calculation of the BCIP Score

GSE51057 (including 177 blood samples from normal women and 146 blood samples from women diagnosed with BC after sample donation) and GSE57285 (including 49 blood samples from normal women and 35 blood samples from women diagnosed with BC after sample donation) were selected to identify differentially methylated probes (DMPs) that correlated with a high risk of BC. In addition, 76 cases with matched tumor and tumor-adjacent breast tissues from TCGA database were enrolled to identify DMPs in BC tissues. CpG probes that were commonly demethylated in blood samples from women with high risk of BC and BC tissues were defined as BCIPs.

DMPs were identified using the R package limma. Differential hyper/hypo-methylation probe was defined according to logFc value. Hyper-methylation probe are defined as logFc >0, *p* value <0.05 (blood/cancer sample of BC patients VS blood/cancer sample of non-BC patients). Hypo-methylation probe are defined as logFc <0, *p* value <0.05 (blood/cancer sample of BC patients VS blood/cancer sample of non-BC patients). The distribution of BCIPs on chromosomes, CpG islands, and TSS regions was investigated using the R package Champ. The hazard ratios (HRs) of BCIPs with respect to OS were evaluated using TCGA-BRCA data.

Univariate Cox regression was used to calculate the HR of each BCIP, and BCIPs significantly correlated cancer survival in BC were included to develop the BCIP score model. The BCIP score model was assessed as follows: BCIP score = [(transformed HR^1^ *β value of BCIP^1^) + (transformed HR^2^ *β value of BCIP^2^) + ······ (transformed HR^n^ *β value of BCIP^n^)]/[abs (transformed HR^1^) + abs (transformed HR^2^) + ······ (transformed HR^n^)]. The cutoff value of BCIP score was 0.2, identified using x-tile (https://medicine.yale.edu/lab/rimm/research/software/). Samples with a BCIP score of <0.2 were assigned to the low BCIP group, while those with a BCIP score of ≥0.2 were assigned to the high BCIP group.

### Functional and Clinical Characteristics Analysis of the BCIP Score in BC

BCIP scores of TCGA-BRCA tissues (76 cases with matched tumor and tumor-adjacent breast tissues and 699 cases with unmatched tumor tissues) were calculated using DNA methylation data. The correlation between the BCIP score and BC-related characteristics (tumor size, oncogene copy number variation, and oncogene expression) was assessed using linear regression and Spearman’s correlation coefficient. The correlation between gene mRNA expression and the BCIP score was analyzed using Spearman’s correlation. The gene expression profile associated with BCIP score was identified based on Spearman’s coefficient (cutoff value: 0.1), and functional study of the related gene expression was performed using the GO and KEGG databases. The above procedure was performed using R software.

### Correlation Analysis Between the BCIP Score and Immune Microenvironment in BC

The degree of infiltration of immune cells and stromal cells in each sample was assessed using the ESTIMATE algorithm (Yoshihara et al., 2013). The proportion of 22 immune cells in each tissue was evaluated using the CIBERSORT algorithm (http://cibersort.stanford.edu/) (Gentles et al., 2015). Correlations between the BCIP score and ESTIMATE and CIBERSORT scores were assessed using linear regression and Spearman’s correlation coefficient.

### Correlation Analysis Between the BCIP Score and Cancer Immunotherapy Response

Four biomarkers were used to assess the response to immunotherapy—CD274, CD8, IFN-*γ* signature (IFNG) (Ayers et al., 2017), and IFNG hallmark gene set (IFNG.GS) (Benci et al., 2019). Three biomarkers were used to assess resistance to immunotherapy—IFN-stimulated gene resistance signature (ISG.RS) (Benci et al., 2019), myeloid-derived suppressor cells (MDSCs), and cancer-associated fibroblasts (CAFs) (Joyce and Fearon, 2015). IFNG was calculated by averaging six genes (IFNG, STAT1, IDO1, CXCL9, CXCL10, and HLA-DRA). IFNG. GS was calculated as previous reported (Benci et al., 2019). CD274, CD8, ISG. RS, MDSCs, and CAFs were assessed using a web application (http://tide.dfci.harvard.edu).

### Establishment and Validation of the Nomogram

A total of 587 BC cases with DNA methylation data, clinical characteristics, and complete follow-up in TCGA database were enrolled as the training cohort, while 231 BC cases with data of DNA methylation, clinical characteristics, and complete follow-up in the GSE72308 dataset were selected as the external validation cohort. The clinical and pathological characteristics of patients with BC are listed in Table 1. Tumor size was defined as ≤2 cm or >2 cm. Lymph node status was defined as non-metastasis or lymph node metastasis. The molecular subtypes were defined based on the IHC assessment of ER, PR, and HER2 as follows: luminal A/B (ER+/PR+/HER2−, ER−/PR+/HER2−, ER+/PR−/HER2−, ER+/PR+/HER2+, ER-/PR+/HER2+, ER+/PR-/HER2+), HER2 (ER−/PR−/HER2+), and TNBC (ER−/PR−/HER2−). Age was defined as 0–39 and ≥40 years.

**TABLE 1:** Characteristics of patients in the training and validation cohort.

Variate	Training cohort (*n* = 587) Num (%)	Validation cohort (*n* = 231) Num (%)
Age		
0–39	37 (6.3)	28 (12.1)
≥40	550 (93.7)	203 (87.8)
Tumor size		
≤2 cm	154 (26.2)	117 (50.6)
>2 cm	433 (73.8)	114 (49.4)
Lymph node status		
Non-metastasis	261 (44.5)	130 (56.3)
Metastasis	326 (55.5)	101 (43.7)
Subtype (IHC)		
Luminal A/B	504 (85.9)	111 (48.1)
HER2	16 (2.7)	55 (23.8)
TNBC	67 (11.4)	65 (28.1)
BCIP score		
Low	425 (72.4)	146 (63.2)
High	162 (27.6)	85 (36.8)
Survival status		
Alive	536 (91.3)	189 (81.8)
Dead	51 (8.7)	42 (18.2)

BC features, which were significantly correlated with BC survival in the multivariate Cox regression, were selected to establish the nomogram model. The patients’ survival probability was assumed by summing the scores of the variates, with a higher score corresponding to a higher mortality risk. The efficiency of the model was evaluated with regard to discrimination and calibration. The concordance index (c-index) was used to quantify discrimination ranging from 0 to 1 (<0.5, absolute discordance; 0.5, equal concordance to chance; and 1, best concordance). The calibration curve was used to compare the predicted survival probability with the observed survival probability at 3, 5, and 10 years in the training and validation cohorts.

## Results

### Identification of BCIPs

**FIGURE 1:**
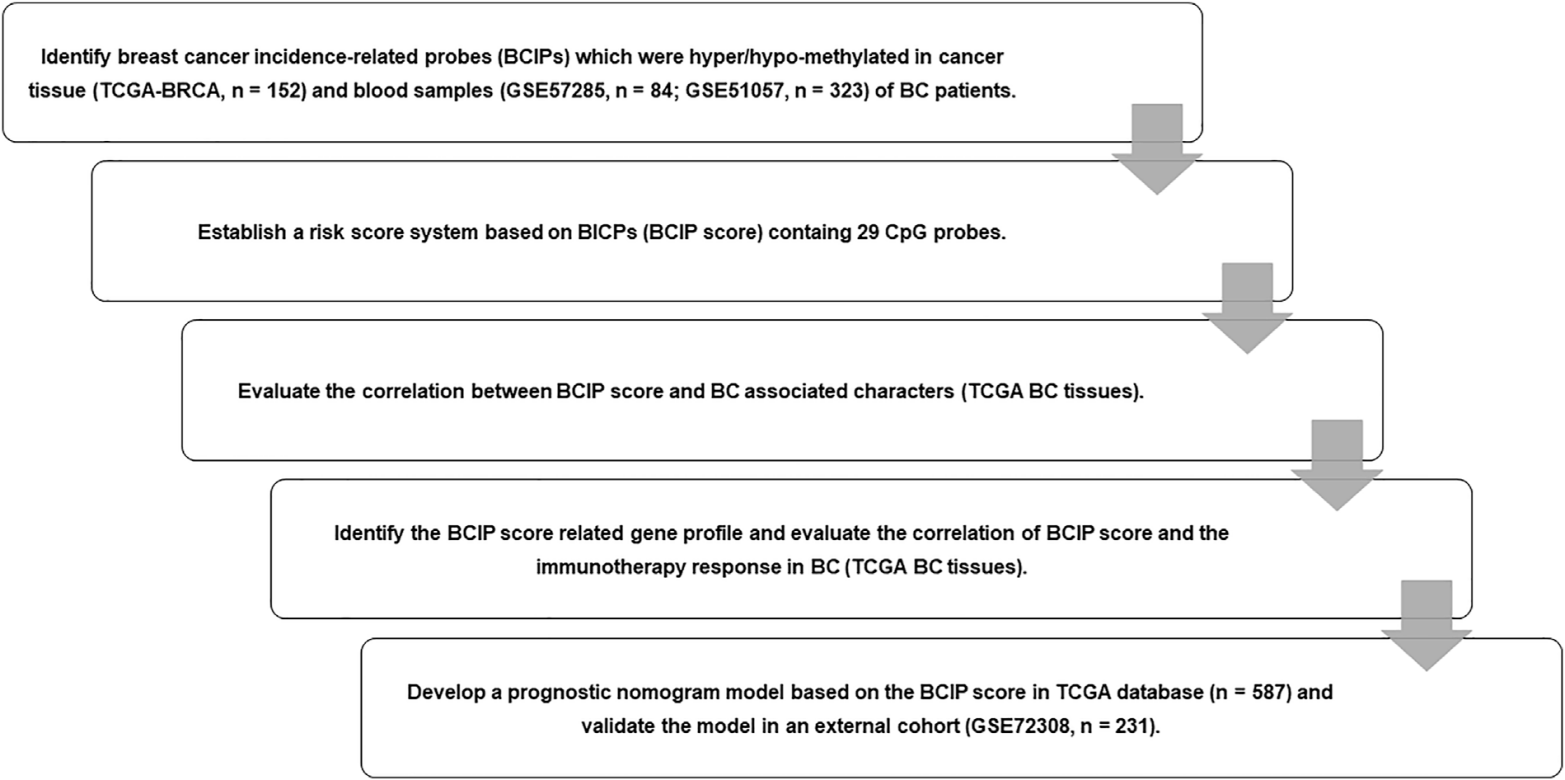
Flow chart of study design. We identified BCIPs that were commonly hypermethylated or hypomethylated in blood samples and cancer tissues in patients with BC; 29 BCIPs that significantly correlated with BC patient survival were selected to develop the BCIP score. The correlation between the BCIP score and clinical characteristics of BC was assessed. We then evaluated the BCIP score-related gene profile and the relationship between the BCIP score and tumor immune response in BC tissues. Furthermore, we assessed the prognostic effect of the BCIP score and developed a prognostic prediction model based on the BCIP score using TCGA database. The efficacy of the prognostic prediction model was validated using the GEO cohort.

**FIGURE 2:**
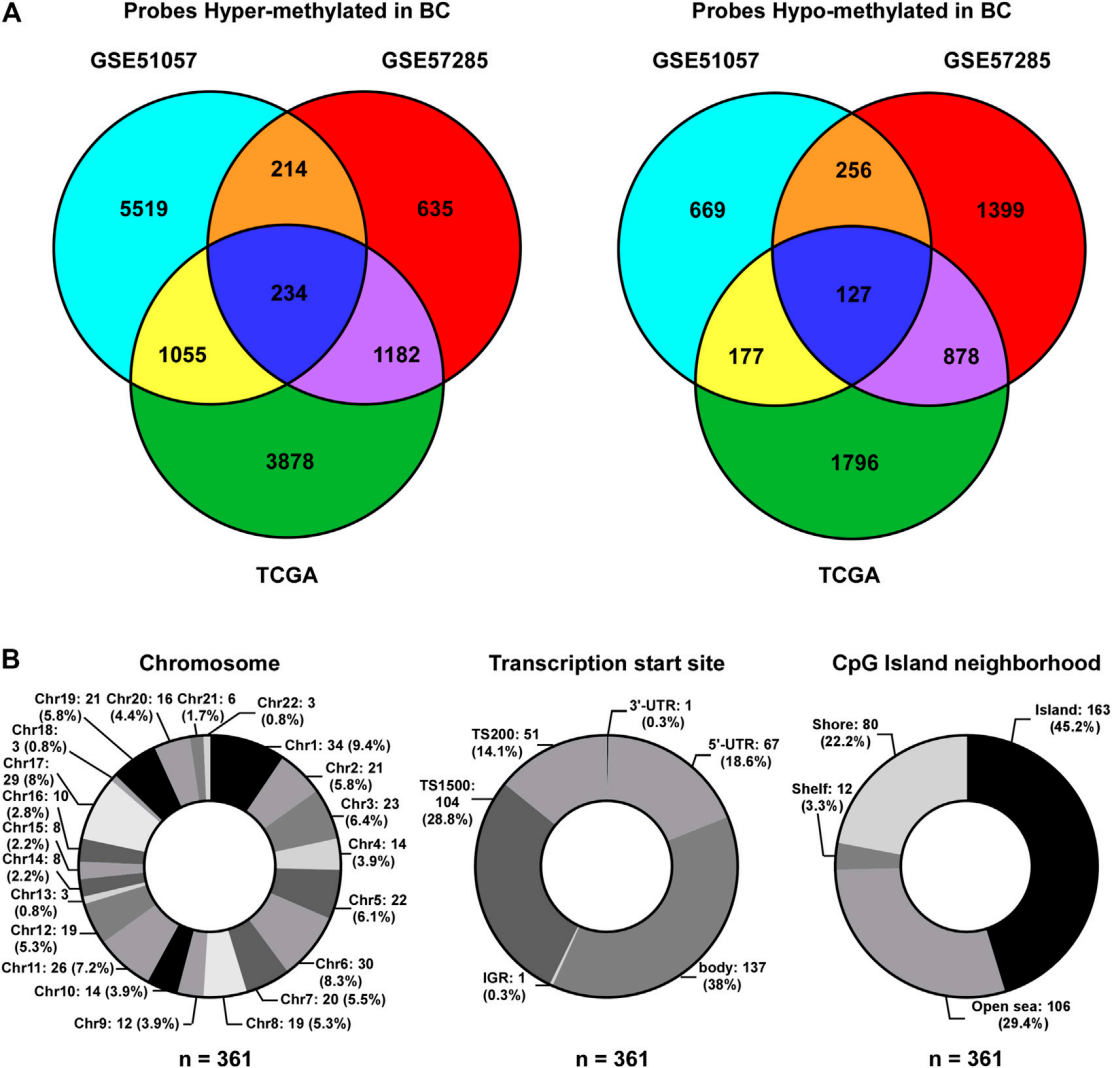
Identification of BCIPs. **(A)** Venn diagram of probes commonly hypermethylated (left panel) or hypomethylated (right panel) in blood samples and cancer tissues from patients with BC. For the GSE51057 and GSE57285 datasets: cases diagnosed with BC vs. normal cases; for TCGA: BC tissues vs. tumor-adjacent tissues. **(B)** Distribution of BCIPs referring to **(Left)** chromosome, **(Middle)** transcription Start Sites, CpG island neighborhood are listed as number (proportion).

To identify CpG probes associated with BC incidence, two GEO datasets (GSE51057 and GSE57285), including 226 blood samples from healthy women (177 cases in GSE51057 and 49 cases in GSE57285) and 181 blood samples from women diagnosed with BC after sample donation (146 cases in GSE51057 and 35 cases in GSE57285; Figure 1), were used. The 448 and 383 CpG probes were commonly hypermethylated or hypomethylated in samples from women with BC compared to those in samples from healthy women (Figure 2A). Further, we included 76 paired tumor-adjacent breast tissues and tumor tissues from TCGA-BRCA database and identified 9,327 differentially methylated probes (tumor vs. tumor-adjacent; hypermethylated: 6,349; hypomethylated: 2,978). By integrating the results from the GEO and TCGA cohorts, 234 hypermethylated and 127 hypomethylated CpG probes were identified as BCIPs in blood samples from women with high risk of BC and BC tissues (Figure 2A). Next, we assessed the distribution of BCIPs based on chromosomes, transcription start sites, and CpG islands. Among the 22 pairs, chromosomes (Chr) 1, 6, 11, and 17 were the most common regions for BCIP distribution; 45.2% were distributed in CpG islands and 42.9% were distributed in the promoter regions (TS200 and TS1500; Figure 2B).

### Establishment of a Prognostic Risk Score Based on BCIPs for BC

**FIGURE 3:**
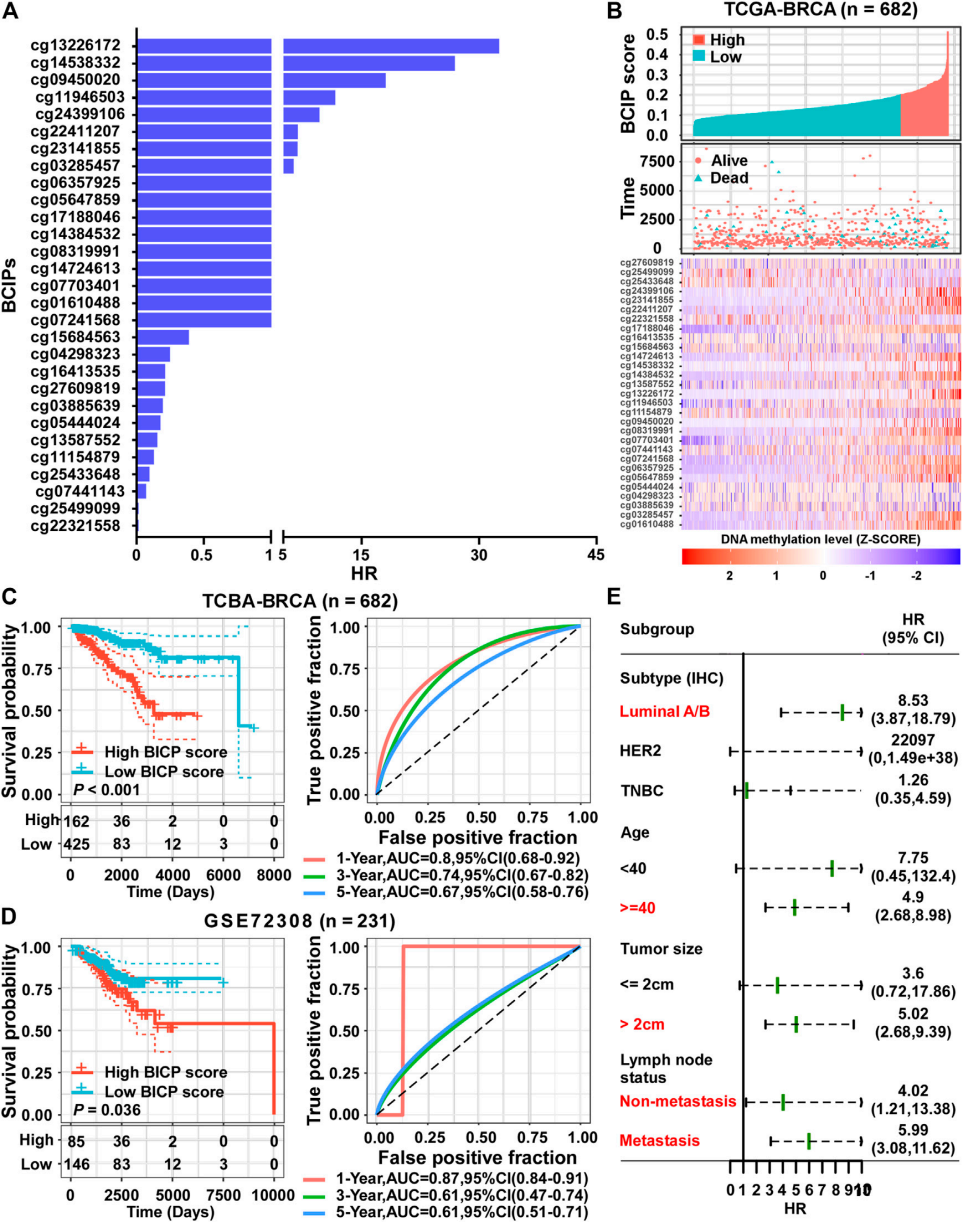
Establishment of a prognostic risk score based on BCIPs for BC. **(A)** HRs of BCIPs were calculated using univariate analysis in TCGA-BRCA cohort (*n* = 682). **(B)** Distribution of BCIP scores (upper panel) in the low-score and high-score subgroups, the death incidence of patients (middle panel), and heatmap of the 29 BCIPs methylation profiles (lower panel) in TCGA cohort. The cutoff value of the BCIP score was identified using x-tile and determined to be 0.2. The DNA methylation levels of BCIPs were normalized using z-score. **(C)** (left panel) Survival analysis of BCIP scores and (right panel) survival prediction ROC curve of the BCIP score in TCGA cohort. **(D)** (left panel) Survival analysis of the BCIP score and (right panel) survival prediction ROC curve of the BCIP score in the GSE72308 cohort. **(E)** Forest plot depicting HRs of BCIP scores in subgroups of TCGA cohort. In subgroups labeled in red, BCIP scores were significantly correlated with overall survival of BC patients. For **(C–D)** (left panel), *p*-values were determined using log-rank test.

Using a univariate Cox regression model, we evaluated the association between methylation levels of BCIPs and overall survival in TCGA-BRCA cohort. DNA methylation levels of 29/361 BCIPs significantly correlated with OS in BC, and these probes were selected to establish the BCIP score model (Figure 3A and Supplemental Table S1). Using X-Tile analysis, the cutoff value for the BCIP score was set at 0.2; patients with a BCIP score ≤0.2 were assigned to the low score group, while patients with a BCIP score of >0.2 were assigned to the high score group (Figure 3B). In TCGA cohort, patients with low BCIP scores had better survival rates than those in the high score group (Figure 3C). The ROC curve showed that the BCIP score exhibited a high predictive efficacy for OS in BC (Figure 3C). Likewise, a high BCIP score predicted a high mortality risk for BC in the GEO cohort (Figure 3D). Further, subgroup analysis indicated that the BCIP score was a negative prognostic factor in subgroups of BC with luminal A/B subtype, old age (≥40 years), larger tumor size, and lymph node metastasis status (Figure 3E and Supplemental Table S2). These data show that the BCIP score is an efficient prognostic model for BC.

### Functional and Clinical Characteristic Analysis of the BCIP Score in BC

**FIGURE 4:**
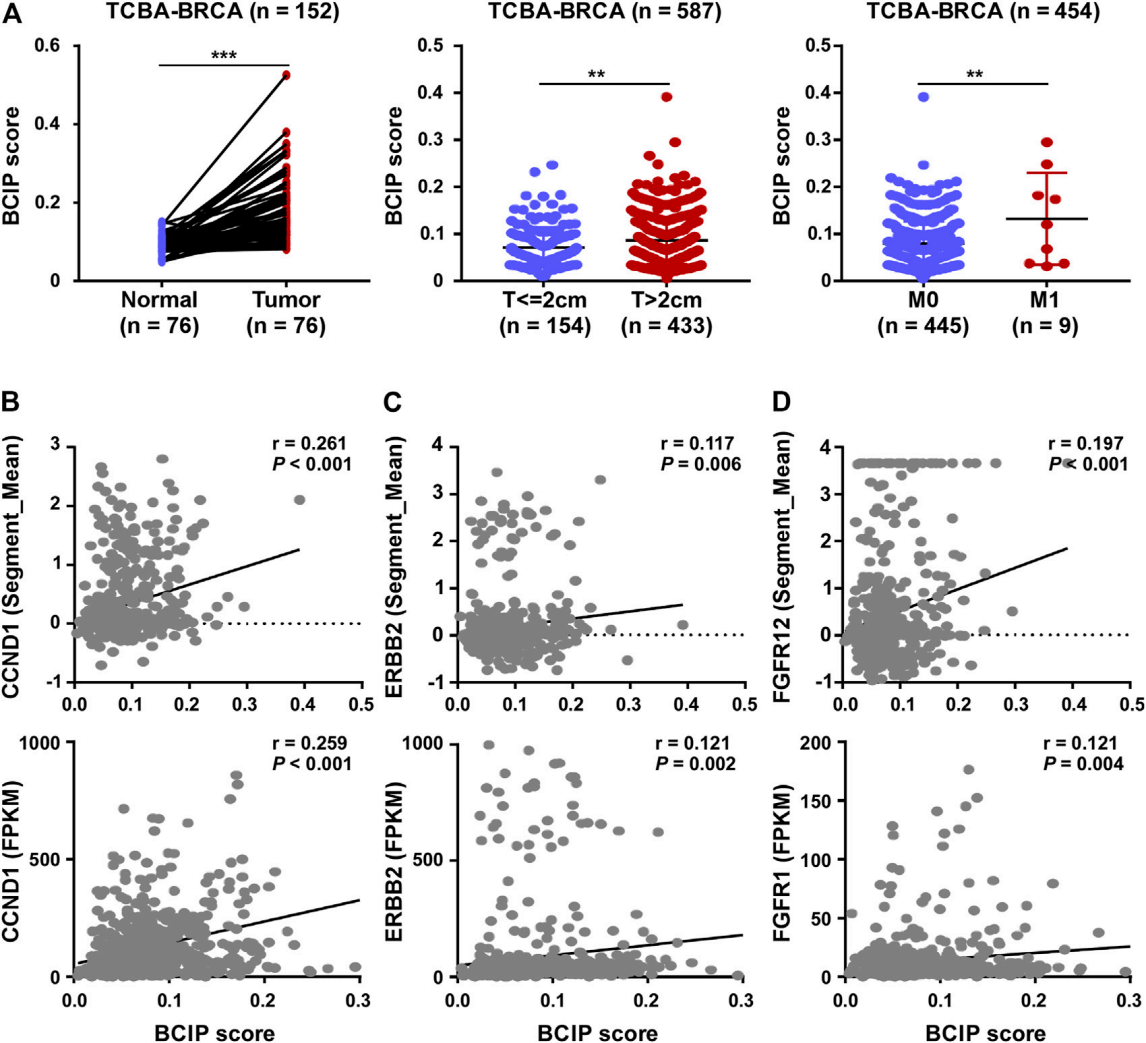
Correlation of BCIP score with BC related characteristics. **(A)** Analysis of BCIP score differences between (left panel) tumor tissues and matched tumor-adjacent breast tissues; (middle panel) cases with tumor size ≤2 cm and cases with >2 cm (right panel) cases with local regional disease (M0) and cases with *de novo* metastasis disease (M1) in TCGA-BRCA cohort. **(B)** Correlations between the BCIP score and (upper panel) copy number and (lower panel) mRNA expression of CCND1. **(C)** Correlations between the BCIP score and (upper panel) copy number and (lower panel) mRNA expression of ERBB2. **(D)** Correlations between the BCIP score and (upper panel) copy number and (lower panel) mRNA expression of FGFR1. For A (left panel), *p*-values were determined by paired *t*-test; for A (middle and right panel), *p*-values were determined by *t*-test; for **(B–D)**, *p*-values were determined by r and Spearman’s correlation coefficient.

Compared to tumor-adjacent breast tissues, BC tissues exhibited a significantly higher BCIP score (Figure 4A). In BC, patients with a larger tumor size and *de novo* metastatic disease had a higher BCIP score (Figure 4A). By assessing the gene copy number in BC tissues, the BCIP score was associated with an increased copy number of several oncogenes (including *CCND1*, *ERBB2*, and *FGFR1*; Figures 4B–D). Consistently, the BCIP score was positively correlated with the mRNA levels of *CCND1*, *ERBB2*, and *FGFR1* (Figures 4B–D).

**FIGURE 5:**
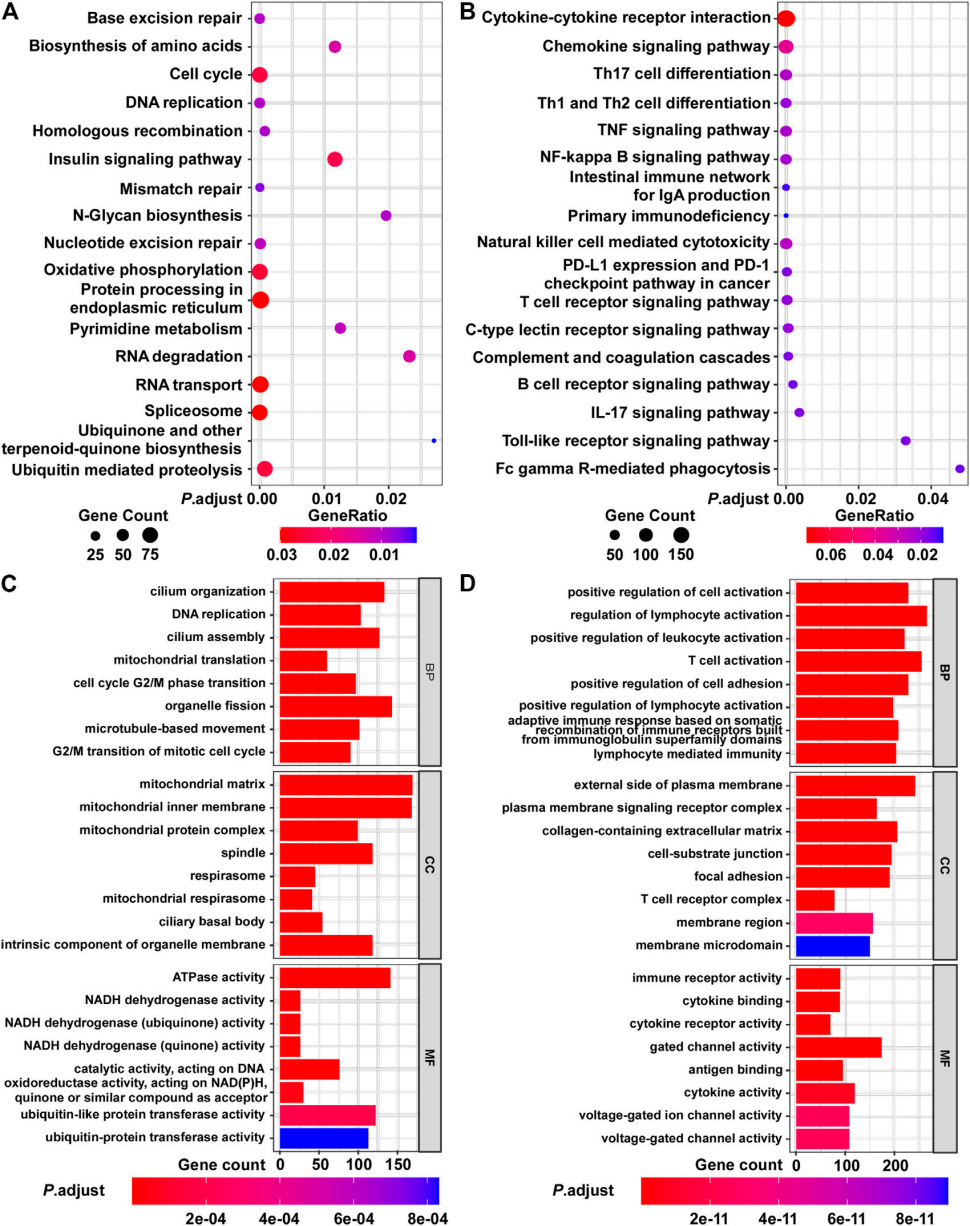
Functional analysis of BCIP score-related gene profile in BC. **(A,B)** Analysis of BCIP score-related gene enrichment in the KEGG pathway. **(A)** Analysis of genes whose mRNA expressions were positively correlated with BCIP score; **(B)** analysis of genes whose mRNA expressions were negatively correlated with BCIP score. Gene ratio was defined as the number of genes enriched to target pathway/number of BCIP score-related gene included in the KEGG dataset. **(C,D)** GO function analysis of BCIP score-related gene. **(C)** Analysis of genes whose mRNA expressions were positively correlated with the BCIP score. **(D)** Analysis of genes whose mRNA expressions were negatively correlated with the BCIP score.

Next, we evaluated the biological significance of the BCIP score in BC. By assessing the correlation between the BCIP score and gene expression, we identified the BCIP score-related gene expression profile. The results of correlation analysis are shown in Supplementary Table S3. Genes with expression correlated with the BCIP score were included in GSEA analysis using the KEGG and GO databases. KEGG and GO analyses showed that genes with expression that positively correlated with the BCIP score were significantly enriched in pathways involving cell cycle regulation, DNA replication, DNA repair (base excision repair, mismatch repair, nucleotide excision repair, and homologous recombination), and energy metabolism (Figures 5A,C). Interestingly, both KEGG and GO analyses showed that genes with expressions that negatively correlated with the BCIP score were significantly involved in cancer-immunity-related pathways (including antigen binding, T cell differentiation and activation, the PD-1 checkpoint pathway, NK cell-mediated cytotoxicity, and immune receptor activity) (Figures 5B,D).

### Correlations Between the BCIP Score and Immune Microenvironment and ICI Response in BC

**FIGURE 6:**
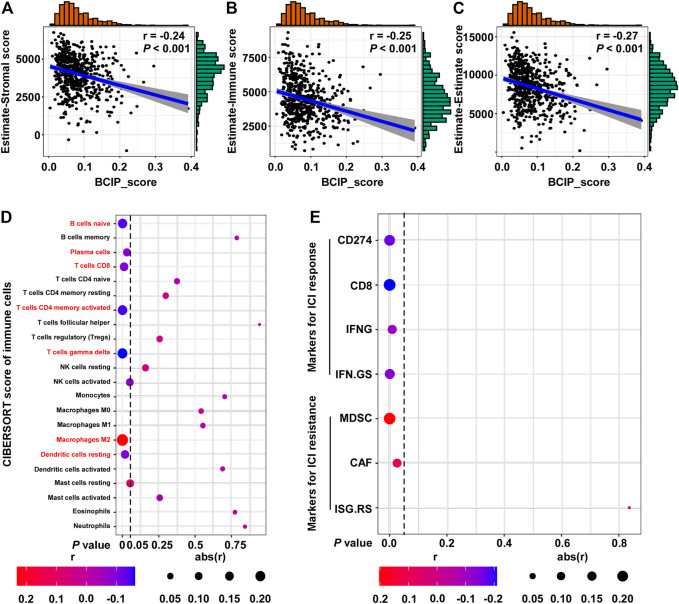
Correlations between the BCIP score and immune microenvironment and ICI response in BC. **(A)** Correlation between the BCIP score and the level of stromal cells (estimate-Stromal score) in BC tissues (TCGA-BRCA, *n* = 587). Numerical distribution of BCIP scores and estimate-Stromal scores are shown on the above the *x*-axis and on the right of the *y*-axis, respectively. **(B)** Correlation between the BCIP score and the level of stromal cells (estimate-Immune score) in BC tissues (TCGA-BRCA, *n* = 587). Numerical distribution of BCIP scores and estimate-Immune scores is shown on the above the *x*-axis and on the right of the *y*-axis, respectively. **(C)** Correlation between the BCIP score and the level of stromal cells (estimate-Estimate score) in BC tissues (TCGA-BRCA, *n* = 587). Numerical distribution of BCIP scores and estimate-Estimate scores are shown on the above the *x*-axis and on the right of the *y*-axis, respectively. **(D)** Correlation between the BCIP score and the 22 type of immune cell components is shown by dot plot. Cell contents correlated with the BCIP score are labeled in red. **(E)** Correlation between DM-BMI and markers for ICI response/resistance is shown by dot plot. r, Spearman’s correlation coefficient.

Further, we used the ESTIMATE algorithm to evaluate the correlation between the BCIP score and degree of immune cell infiltration in BC tissues. The BCIP score was significantly correlated with decreased levels of immune and stromal cell content, indicating that the BCIP score was negatively correlated with immune cell infiltration in BC tissues (Figures 6A–C). The CIBERSORT algorithm was used to evaluate the association between the BCIP score and 22 immune cell contents in BC tissues (TCGA-BRCA cohort). The BCIP score was negatively correlated with the level of several immune cells with antitumor activity (including plasma, CD8^+^ T, CD4^+^ T, and gamma delta T cells; Figure 6D). In addition, the level of resting dendritic cells, which play a critical role in antigen phagocytosis and processing, decreased in BC tissues with increased BCIP scores (Figure 6D). In contrast, the level of M2 macrophages, which promote tumor progression, was positively correlated with the BCIP score (Figure 6D). These results indicate that the BCIP score was correlates with poor antitumor immunity. As a previous study showed that ICIs (Shah et al., 2012) significantly improved cancer survival through T cell immunity in BC, we further investigated whether the BCIP score correlated with the ICI response in BC. Four markers for ICI sensitivity and three markers of ICI resistance were selected to evaluate the ICI response in BC tissues. The BCIP score was negatively correlated with ICI-sensitive markers (CD274, CD8, IFNG, and IFN. GS; Figure 6E). In contrast, two of the three ICI resistance markers (MDSC and CAF) were positively correlated with the BCIP score in BC (Figure 6E). Collectively, the BCIP score was a negative marker of immune cell infiltration and ICI response in BC.

### Establishment of a BCIP Score-Based Nomogram Model to Predict Overall Survival in BC

**FIGURE 7:**
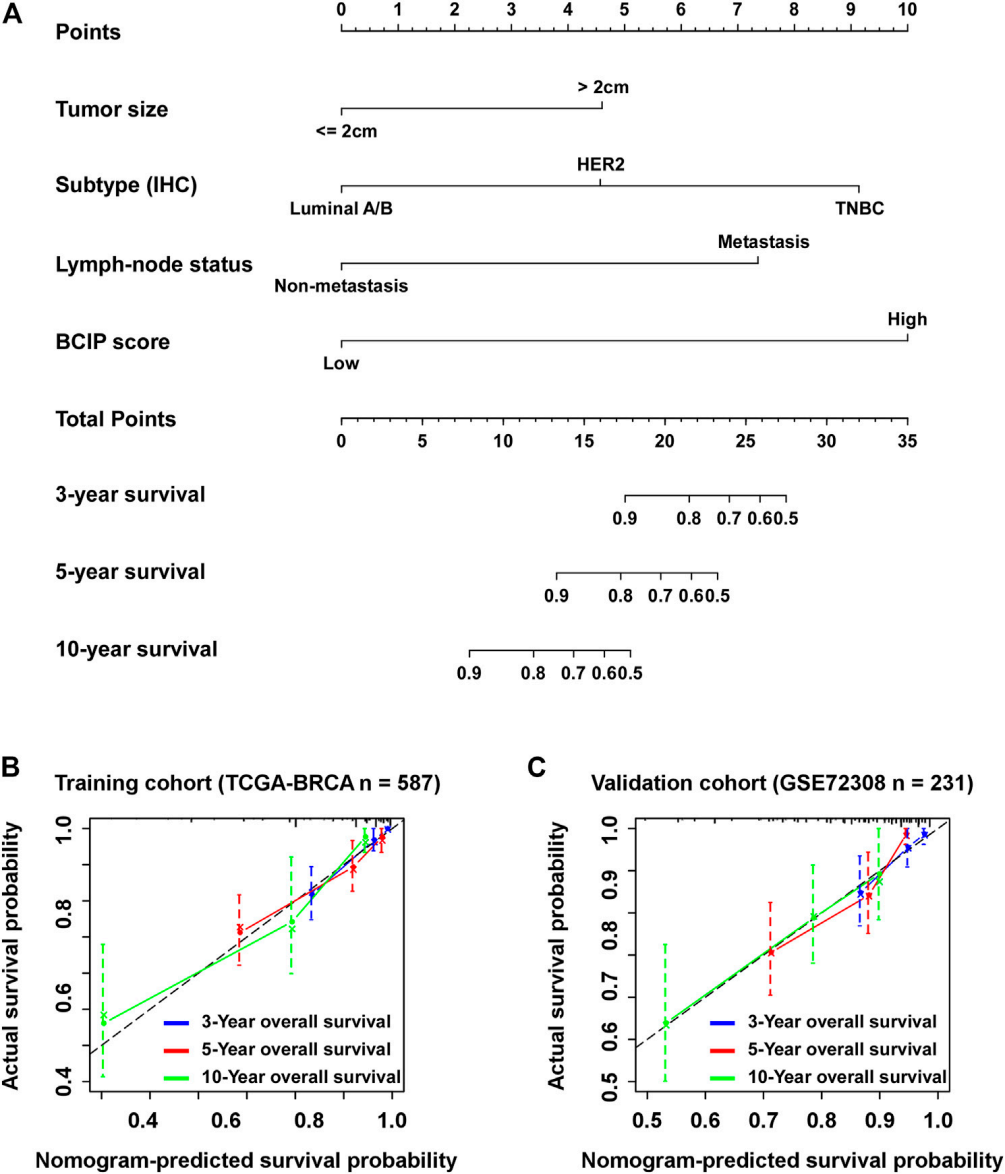
Establishment of the BCIP score-based nomogram model to predict overall survival in BC. **(A)** Prognostic nomogram for patients with BC with factors including tumor size, molecular subtype, lymph node status, and the BCIP score. Points are defined based on the prognostic contribution each factor. Points summing the contribution of tumor size, molecular subtype, lymph node status, and the BCIP score are translated to the survival probability at 3, 5, and 10 years **(B,C)** Calibration plots for predicting patient overall survival at 3, 5, and 10 years in **(B)** TCGA and **(C)** the GSE72308 cohorts. Probability of survival based on the nomogram is listed on the *x*-axis, while the actual probability of survival is listed on the *y*-axis.

Univariate and multivariate Cox regression analyses showed that tumor size, molecular subtype, lymph node status**,** and the BCIP score were significant prognostic factors for BC (Table 2). After including the above variables, we developed a comprehensive prognostic nomogram based on TCGA-BRCA cohort (Figure 7A). Factors correlated with high mortality risk (larger tumor size, TNBC subtype, metastatic lymph node status, and high BCIP score) were scored higher than those correlated with low mortality risk (smaller tumor size, luminal A/B subtype, non-lymph node metastasis, and low BCIP score). The c-index of this model was 0.831 (95% CI: 0.774–0.888) in TCGA-BRCA cohort. Furthermore, data from the GSE72308 dataset were selected for external validation of the nomogram. The c-index of the model was 0.734 (95% CI: 0.665–0.803) in the external validation cohort. The calibration curve was constructed to evaluate the accuracy of model prediction and indicated that the BCIP score-based nomogram exhibited good consistency in the prediction of 3-, 5-, and 10-year survival probabilities in both TCGA-BRCA and the GSE72308 cohorts (Figures 7B,C).

**TABLE 2:** Univariate and multivariate analysis for patients with BC in TCGA cohort.

Variate	Univariate			Multivariate		
	HR	95% CI	*p* Value	HR	95% CI	*p* Value
Age			0.339			
Age	1					
0–39	1.799	0.54–5.989				
≥40			0.04			0.045
Tumor size	1			1		
≤2 cm	2.467	1.044–5.83		2.41	1.021–5.688	
>2 cm			<0.001			<0.001
Lymph node status	1			1		
Non-metastasis	4.18	2.087–8.371		3.959	1.997–7.848	
Subtype (IHC)						<0.001
Luminal A/B	1		<0.001	1		
HER2	3.39	0.789–14.561		3.445	0.801–14.816	
TNBC	6.034	3.016–12.073		5.561	2.833–10.918	
BCIP score			<0.001			<0.001
Low	1			1		
High	6.618	3.628–12.073		6.748	3.701–12.304	

## Discussion

For years, prognostic prediction in patients with BC has been mostly based on the pathological features of the tumor (including tumor size, lymph node status, distant metastatic status, and molecular subtype) (Harbeck and Gnant, 2017). BC therapy guided by these prognostic indicators has significantly improved cancer survival and avoids the therapeutic side effects caused by overtreatment in BC (DeSantis et al., 20192019). With advances in BC treatment, more precise prognostic methods are required (Denkert et al., 2017). Several prognostic biomarkers, including gene sequencing, gene copy number, and circulating tumor cells, have been used in clinical practice for BC (Lord and Ashworth, 2016; Tsai et al., 2018; Radovich et al., 2020). For instance, the 21-gene assay (Oncotype Dx) has been used to identify patients with low recurrence risk who could be exempted from chemotherapy in T1b/c and T2, HR+, HER2-, and lymph node-negative BC (Giuliano et al., 2017). Detection of *BRCA1/2* mutation status is helpful in identifying the potential benefits of PARP inhibitors (Tarsounas and Sung, 2020). Recently, Fackler et al. identified hypermethylation signatures that were correlated with cancer recurrence in TNBC (Fackler et al., 2020). In this study, we identified DNA methylation signatures related to BC incidence and developed a nomogram based on DNA methylation to calculate the 3-, 5-, and 10-year survival probabilities for BC.

An increasing amount of data has shown that aberrations in DNA methylation correlated with breast malignancy development is a prerequisite for the transformation of normal cells into BC cells, and these changes in DNA methylation accumulate in malignant cells, inducing an enhanced ability for proliferation and self-renewal (Zhuang et al., 2012; Wienken et al., 2016). As DNA methylation is regulated by environmental factors, genetic predisposition, and individual lifestyle, variation in DNA methylation pattern can be a reflection of the individual response to exposure to BC risk (Widschwendter et al., 2018). Identification of cancer incidence-related DNA methylation pattern is of great value for prognostic prediction in BC. Hence, we developed the BCIP score based on BC-related DNA methylation pattern and observed that the BCIP score was significantly correlated with poor outcomes in patients with BC.

In BC tissues, the BCIP score not only acted as a prognostic biomarker but also significantly correlated with aggressive BC features (such as larger tumor size, distant metastatic disease, and oncogene amplification). In addition to being a reflection of biological changes, changes in DNA methylation play a critical role in tumor progression through the regulation of gene expression. By assessing the BCIP score-related gene expression profile, we discovered that genes with expressions that were negatively correlated with the BCIP score were also significantly involved in the cancer immunity-related pathway. As a high BCIP score correlated with increased mortality, an aberration of cancer immunity might account for the poor outcome of patients with a high BCIP score.

In our study, we observed that BCIP scores were negatively correlated with the degree of T cell infiltration in BC tissues, indicating that the T-cell-mediated immune response (cellular immunity) was aberrantly inactivated. Cellular immunity is the main type of tumor immune response, which is mediated by the direct killing effect of T cells and release of cytokines by T cells (Joyce and Fearon, 2015). When activated by tumor antigens, T cells become immunoreactive and acquire the ability to recognize and kill tumor cells. However, T cell activation is often blocked by immune checkpoint molecules (including PD-1/PDL1, CTLA-4, and TIM-3) in individuals with cancer (Joyce and Fearon, 2015; Voorwerk et al., 2019). In BC, ICIs targeting PD1/PDL1 have been proven to improve survival in some patients, while most patients exhibit a poor response to ICIs (Voorwerk et al., 2019). To improve the efficacy of ICIs, effective biomarkers are required to identify potential beneficiaries. As our data showed that the BCIP score was negatively correlated with ICI response markers, the BCIP score is a potential biomarker to predict the sensitivity of BC to ICIs.

It is well known that DNA methylation correlates with suppressed gene expression, indicating that DNA methylation alterations can be exploited in cancer diagnosis. Compared with other genetic approaches (such as mutational analysis), DNA methylation-based approaches present advantages with regard to their clinical application (Heyn and Esteller, 2012). For instance, DNA methylation detection mostly focuses on a specific promoter region containing CpG islands, while mutational studies can cope with large regions since point mutations are located throughout the length of the gene (Heyn and Esteller, 2012). Moreover, alterations in DNA methylation were detected in a higher proportion of tumor tissue than genetic alterations, leading to higher sensitivity in prognostic analysis (Esteller et al., 2001). In our study, we developed a BCIP scoring system based on BC-related DNA methylation variation and observed that the BCIP score was positively correlated with mortality in patients with BC. Further, we adjusted for the confounding effects of tumor size, lymph node status, and molecular subtypes using Cox regression and found that BCIP was an independent prognostic factor for BC. By integrating the BCIP score, tumor size, lymph node status, and molecular subtypes, we established a nomogram model that could accurately quantify the survival probability of patients with BC. Based on our nomogram, patients with BC and a low mortality risk could be identified and might be exempted from aggressive and excessive medical treatment. However, patients with a high mortality risk should undergo more intensive surveillance for cancer recurrence.

Collectively, our study identified the variation in DNA methylation pattern related to BC incidence and developed a BCIP score model depicting BC-related DNA methylation variation. In BC, the BCIP score was significantly correlated with malignant BC characteristics and poor outcomes. Furthermore, we observed that the BCIP score was negatively correlated with immune cell infiltration and ICI response markers in BC tissues. Finally, a nomogram comprising the BCIP score, tumor size, lymph node status, and molecular subtype was developed to quantify the survival probability of patients with BC.

## Data Availability

The original contributions presented in the study are included in the article/Supplementary Material, further inquiries can be directed to the corresponding authors.

## Abbreviations

BC, breast cancer; BCIPs, breast cancer incidence-related probes; c-index, the concordance index; CAFs, cancer-associated fibroblasts; Chr, Chromosome; DMP, differentially methylated probe; DNMTs, DNA methyltransferases; knn, k-nearest neighbors; ER, estrogen receptor; GEO, Gene Expression Omnibus; GO, Gene Ontology; GSEA, Gene Set Enrichment Analysis; HRs, hazard ratios; ICI, immune checkpoint inhibitor; IFNG, IFN-*γ* signature; IFNG.GS, IFNG hallmark gene set; IFN.ISGs, IFN stimulated genes resistance signature; KEGG, Kyoto Encyclopedia of Genes and Genomes; MDSCs, myeloid-derived suppressor cells; PR, progesterone receptor; TNBC, triple-negative breast cancer; SNPs, single nucleotide polymorphisms; TCGA, The Cancer Genome Atlas.
